# Case report: Dramatic response to pralsetinib in an elderly patient with advanced RET-fusion positive papillary thyroid carcinoma

**DOI:** 10.3389/fonc.2022.1042525

**Published:** 2022-12-12

**Authors:** Margherita Nannini, Andrea Repaci, Gianluca Ricco, Manuela Ianni, Arber Golemi, Vincenzo Maiolo, Marco Ferrari, Filippo Natali, Elisa Lodi Rizzini, Fabio Monari, Erica Solaroli, Antonio De Leo, Thais Maloberti, Maria A. Pantaleo, Dario De Biase, Giovanni Tallini

**Affiliations:** ^1^Oncology Unit, IRCCS Azienda Ospedaliero-Universitaria di Bologna, Bologna, Italy; ^2^Department of Experimental, Diagnostic and Specialty Medicine, S. Orsola-Malpighi University Hospital, Bologna, Italy; ^3^Division of Endocrinology and Diabetes Prevention and Care, IRCCS Azienda Ospedaliero-Universitaria di Bologna, Bologna, Italy; ^4^ UO Ricerca e Innovazione, Clinical Trials Centre, IRCCS Azienda Ospedaliera Sant'Orsola Malpighi-Università di Bologna, Bologna, Italy; ^5^Nuclear Medicine Division, IRCCS AOU Azienda Ospedaliero-Universitaria di Bologna, Bologna, Italy; ^6^Radiology Uniti, IRCCS Azienda Ospedaliero-Universitaria di Bologna Policlinico S Orsola-Malpighi, Bologna, Emilia-Romagna, Italy; ^7^Interventional Pulmonology Unit, IRCCS Policlinico Sant’Orsola, Azienda Ospedaliero-Universitaria di Bologna, Bologna, Italy; ^8^Radiation Oncology, IRCCS Azienda Ospedaliero-Universitaria di Bologna, Sant'Orsola-Malpighi Hospital, Bologna, Italy; ^9^Endocrinology Unit, Azienda USL of Bologna, Bologna, Italy; ^10^Department of Experimental, Diagnostic and Specialty Medicine, University of Bologna, Bologna, Italy; ^11^Solid Tumor Molecular Pathology Laboratory, IRCCS Azienda Ospedaliero-Universitaria di Bologna, Bologna, Italy; ^12^Department of Pharmacy and Biotechnology (FaBit), University of Bologna, Bologna, Italy

**Keywords:** differentiated thyroid cancer, papillary thyroid carcinoma, RET, RET-rearrangement, RET-inhibitor, pralsetinib

## Abstract

We are recently faced with a progressive evolution of the therapeutic paradigm for radioiodine refractory differentiated thyroid cancer (RAI-R DTC), since the advent of tissue agnostic inhibitors. Thus, tumor genotype assessment is always more relevant and is playing a crucial role into clinical practice. We report the case of an elderly patient with advanced papillary thyroid carcinoma (PTC) harboring *RET-CCDC6* fusion with four co-occurring mutations involving *PI3KCA*, *TP53*, and *hTERT* mutations, treated with pralsetinib under a compassionate use program. Despite the high histological grade and the coexistence of aggressive RET co-mutations, an impressive metabolic and structural tumor response has been obtained, together with a patient’s prolonged clinical benefit. A timely comprehensive molecular testing of those cases wild-type for the common thyroid carcinoma *BRAF* V600E-like and *RAS*-like driver mutations may uncover actionable gene rearrangements that can be targeted by highly selective inhibitors with great potential benefit for the patients.

## Introduction

During the last decades, the biological background of differentiated thyroid cancer (DTC) has come into focus leading to significant therapeutic improvement of metastatic patients with a radioiodine refractory (RAI-R) disease. Indeed, the identification of genetic alterations in thyroid carcinogenesis, mainly involving the *mitogen-activated protein kinase* (*MAPK*) and *phosphatidylinositol 3-kinase* (*PI3K*)/*protein kinase B* (*AKT*) signaling pathways, has been instrumental to explore the role of targeted therapies in DTC, making lenvatinib and sorafenib the current standard-of-care of treatment for metastatic/advanced RAI-R patients (1–4). More recently, therapeutic options of RAI-R DTC have further expanded with the advent of *neurotrophic tyrosine receptor kinase* (*NTRK*) and *RET*-inhibitors, which have shown marked and durable responses in patients with metastatic/advanced RAI-R DTC positive for *NTRK* or *RET* gene fusions, with a favorable safety profile (4–7). In this growing landscape, we are faced with a progressive evolution of the therapeutic paradigm for RAI-R DTC, for which tumor genotype assessment is thus playing crucial role. Herein, we report the case of an elderly patient with advanced papillary thyroid carcinoma (PTC) harboring *RET::CCDC6* fusion with four co-occurring mutations involving *PI3KCA*, *TP53*, and *hTERT* mutations, with an impressive clinical and radiologic response to pralsetinib under a compassionate use program (AG43388 – Roche).

## Case presentation

**Figure 1:**
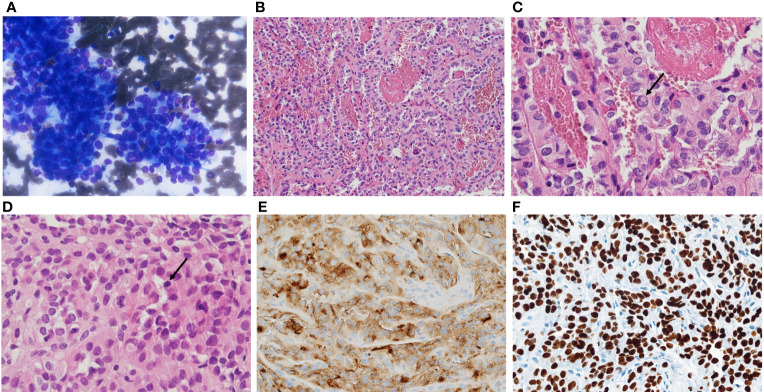
**(A)** fine needle aspiration showing atypical cells forming papillary clusters, diagnosed as TIR5-SIAPEC (Bethesda VI and to Thy5-BTA), compatible with papillary thyroid carcinoma. Histologically the tumor showed complex papillary architecture **(B)** intranuclear inclusions (**C**, arrow), mitotic activity (**D**, arrow), immunohistochemical thyroglobulin **(E)** and TTF1 expression **(F)**.

An 84-years old man, with a history of systemic hypertension, type II diabetes mellitus, ischemic cardiomyopathy and atrial fibrillation (AF) sought medical attention after two months exertional dyspnea and throat tightness. Thus, in August 2020 diagnostic investigations were performed. Neck ultrasonography showed a worrisome highly vascular nodule in the right thyroid lobe (45.5 x 41.1 x 58 mm) and right latero-cervical lymph nodes at III and IV levels (26.1 x 16.1 x 24.6 mm). Thyroid and nodal fine needle aspiration (FNA) cytology identified malignant cells (Bethesda VI) compatible with papillary thyroid carcinoma (**Figure 1A**). Basal levels of thyroid-stimulating hormone (TSH) and thyroglobulin (Tg) were 0.7 mcU/ml and 1026 ng/ml, respectively. A fibrobronchoscopy was performed due to patient’s symptoms, revealing *ab estrinseco* stenosis of the trachea with vegetating neoplastic tissue within the lumen. An endotracheal stent was placed, and the tumor was biopsied. The diagnosis was PTC with high grade features due to the presence of mitotic activity and necrosis (**Figures 1B-D**). Neoplastic cells were positive for Thyroid Transcription Factor- 1 (TTF-1) and Tg (**Figures 1E, F**).

Next-generation sequencing (NGS) analysis - performed on the thyroid cytology specimen using a laboratory-developed multi-gene panel (8) and the Oncomine Focus panel assay – demonstrated *RET:CCDC6* rearrangement with three additional co-occurring mutations involving *PI3KCA* (p.N1044K c.3132T>A, Exon 21), *TP53* (p.Y163C c.488A>G, Exon 5), and *hTERT* (C228T, Promoter).

**Figure 2:**
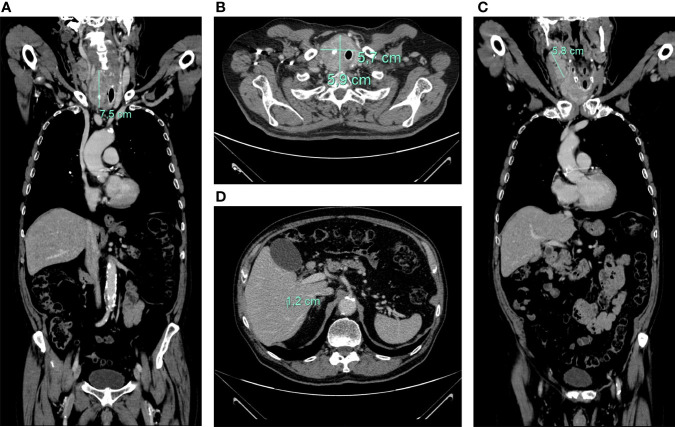
axial **(A)** and coronal **(B)** CT-scan showing large inhomogeneous tumor with calcifications of the right thyroid lobe with left tracheal displacement, infiltrating the trachea and esophagus; confluent neck lymph node metastases (maximum diameter of 58 mm) **(C)**; hepatic lesion of 12 mm at V segment **(D)**.

Total-body computed tomography (TB-CT) scan demonstrated the tumor of the right thyroid lobe dislodging and infiltrating cervical esophagus and trachea, a partially necrotic confluent right latero-cervical lymph node metastases (maximum diameter of 5.8 cm), bilateral non-calcified pulmonary nodules, mediastinal lymph nodes, and a 12 mm liver lesion at V segment (**Figure 2**).

**Figure 3:**
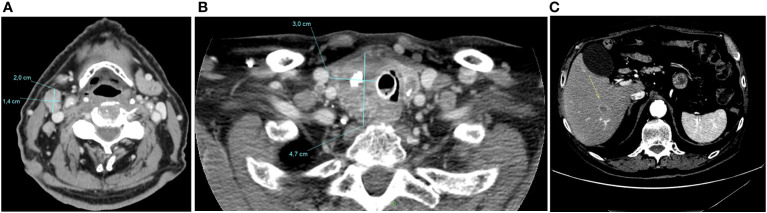
CT-scan performed in March 2021, after four months of Lenvatinib, showing a dimensional reduction of latero-cervical lymphadenopathies (maximum diameter of 2 cm) **(A)**, and of left thyroid lobe (3x4,7 cm) **(B)**, and a mild increase of the hepatic lesion (17 mm vs 12 mm) **(C)**.

Total thyroidectomy and laryngectomy were not performed due to the extent of the disease, the patient’s age and his multiple comorbidities. Thus, in November 2020 systemic therapy with lenvatinib at personalized dose of 14 mg daily was started. The patient experienced several side-effects, such as asthenia grade 2 (G2), stomatitis G2, diarrhea G2, leading to transient treatment interruption and further dose reduction to 10 mg daily. At the first CT-scan evaluation, performed in March 2021, a dimensional reduction of latero-cervical lymphadenopathies (maximum diameter of 2x3 cm), and of left thyroid lobe was obtained, while a mild increase of the hepatic lesion was documented (17 mm vs 12 mm) (**Figure 3**). Thus, lenvatinib was continued until June 2021 when it was stopped due to the onset of severe anemia G4 (Hb 6 gr/dL, MCV 94 fL) from a gastrointestinal bleeding probably related to the concomitant anticoagulant therapy for AF.

**Figure 4:**
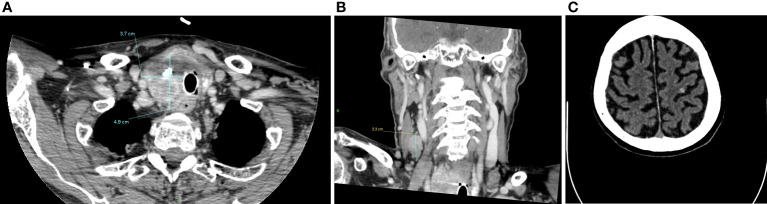
CT-scan performed in August 2021, after lenvatinib discontinuation, showing a dimensional increase of left thyroid lobe (4,9x3,7 cm) **(A)**, latero-cervical lymphadenopathies (maximum diameter of 3 cm) **(B)** and multiple brain and cerebellar metastases **(C)**.

**Figure 5:**
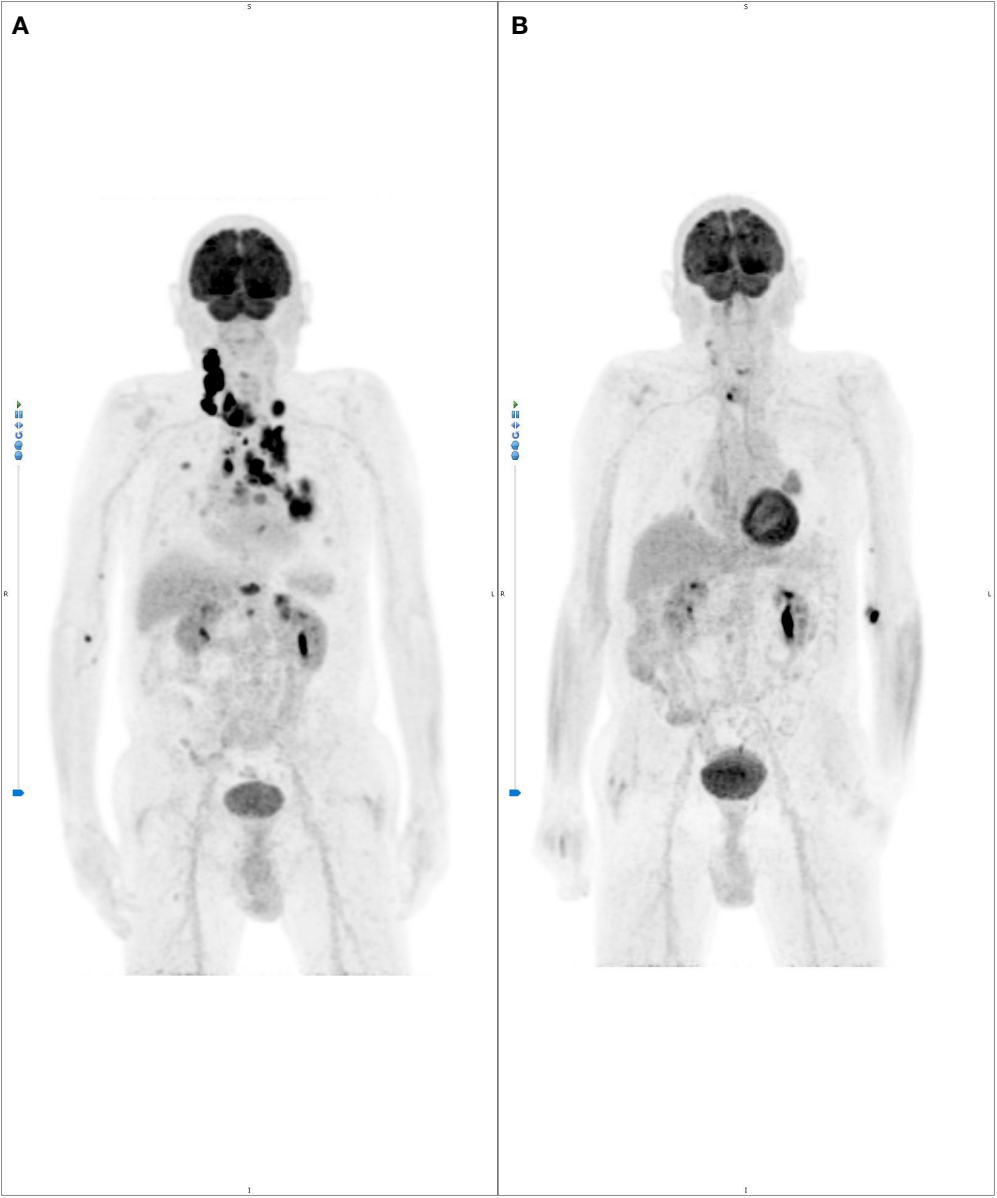
**(A)** basal 18FDG-PET scan showing intense signal in the right thyroid lobe (standardize uptake value, SUV max: 23.7), right neck metastasis (SUV max: 44.5), bilateral supraclavicular lymph nodes (SUV max: 31, right; SUV max: 15., left), lung nodules (Left lower lobe, SUV max: 15.1- Posterior and 17.0-Paravertebral; Right upper lobe, SUV max: 6.5 and 6.2), mediastinal lymph nodes (left pulmonary hilus, SUV max: 10.4; left peribronchial, bilateral tracheo-bronchial and subcarinal, SUV max: 7.3), upper thoracic outlet, paraesophageal, right para-tracheal lymph nodes (SUV max: 18.1); **(B)** remarkably rapid metabolic response within less than one month of pralsetinib therapy.

However, as a consequence of this treatment period-off, in August 2021, a CT-scan was performed due to markedly increased Tg levels (2495 vs 749 ng/ml), reveling disease progression with the onset of at least six bilateral brain and two cerebellar lesions, all with hemorrhagic features (**Figure 4**). Subsequent 18F-fluoro-deoxy-glucose positron emission tomography (18F-FDG-PET) showed high glucose uptake in the right thyroid lobe (SUVmax = 23.7), together with intense uptake at a very large right latero-cervical adenopathy (SUVmax = 44.5), and at several additional lymph nodal and visceral sites, including lung, bone and pancreas (**Figure 5A**).

Given the tumor molecular profile, taking into account the patient’s good performance status and the gastrointestinal bleeding, a second line of treatment with pralsetinib within a compassionate use protocol (AG43388 – Roche) was considered. The study was approved by the local Ethic Committee of S. Orsola-Malpighi Hospital, Bologna (208/2021/Compass/AOUBo) and the patient provided informed consent. Pralsetinib was started in September 2021 at the standard dose of 400 mg daily, and subsequently personalized to 200 mg daily, due to the onset of G3 diarrhea and G2 asthenia. Noteworthy, the 18F-FDG-PET performed after one month of therapy for early treatment response evaluation, reported a complete metabolic response in almost all sites and a relevant partial response in the others (**Figure 5B**). CT-scan performed four months after the beginning of therapy showed dramatic tumor shrinkage of both primary tumor and of all metastatic lesions, including the brain metastases, for which an almost complete radiological response was obtained, with a consequent patient’s clinical benefit especially on dyspnea and throat tightness, and an important drop of Tg levels from 2495 to 485 ng/ml.

**Figure 6:**
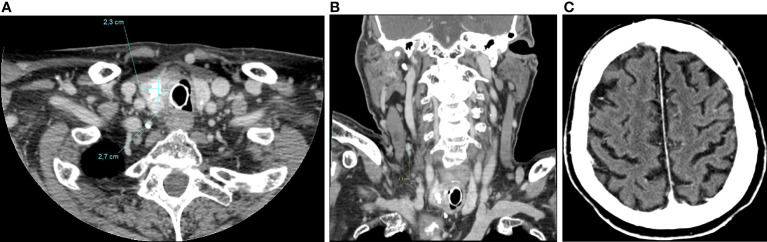
CT-scan performed in October 2022, 13 months after the beginning of treatment, showing a further tumor volume reduction of left thyroid lobe (2,3x2,7cm) **(A)**, of latero-cervical lymphadenopathies (maximum diameter of 1,3 cm) **(B)**, and a complete radiological response of brain metastases **(C)**.

At present the patient is still in treatment with pralsetinib at the personalized dose of 200 mg daily, with an overall good tolerance. The last CT-scan performed in October 2022, 13 months after the beginning of treatment, showed a further tumor volume reduction and Tg levels have drop to 378 ng/ml (**Figure 6**).

## Discussion

*RET* fusion represents the most common gene fusion event in DTC, present in about 10-20% of papillary thyroid carcinoma (9). The reported prevalence of *RET* fusion is very heterogeneous across studies, due to geographic variation, as well as to different sensitivities of detection methods used (10–12). So far, more than 20 fusions of the *RET* tyrosine kinase domain to the 5`-terminal region of heterologous genes have been reported resulting in RET/PTC chimeric oncogenes, of which *CCDC6::RET* (RET/PTC1) and *NCOA4::RET* (RET/PTC3) are the most common types (9, 13, 14).

RET rearrangements consist of the fusion of RET to the 5`-terminal region of partner genes, resulting in RET oncogenes and then into the corresponding fusion oncoproteins. These RET chimeric proteins dimerize, leading to the constitutive kinase activation of RET and to its autophosphorylation. This mechanism results in enhancing signal transduction along classical downstream pathways of both *MAPK* and the *PI3K/AKT* (9, 13).

*RET*-rearranged PTC develops frequently in pediatric and in radiation-exposed patients, whereas in older patients is rarer (15–17). Morphologically, *RET*-rearranged PTC are characterized by extensive lymph-vascular involvement, stromal sclerosis, prominent lymphocytosis, squamous metaplasia, and numerous psammoma bodies, features typical of diffuse sclerosing PTC that is indeed commonly associated with *RET* rearrangement (18–20). Similarly, to most other fusion-positive DTC, they thus share a unique histologic pattern, characterized by the triad of multinodularity, prominent fibrosis, and lymph-vascular invasion (18). Some *RET* fusion-positive PTC are aggressive with extrathyroidal extension, multifocality, lymph node involvement and distant metastases (18, 21–24). In some cases, aggressiveness is strongly associated with the high-grade features as well as *RET* co-alterations (18). Indeed, in the present case co-occurring *PI3KCA*, *TP53*, and *hTERT* mutations likely underlie the high-grade histologic features and the aggressive clinical course of the disease. The variant allele frequencies (VAF) of *hTERT*, *TP53*, and *PI3KCA* mutations (52%, 27%, and 8%, respectively) suggest that they occurred sequentially - in the order reported above - during tumor progression.

In the newborn era of tissue agnostic therapies, the treatment landscape of *RET*-rearranged PTC has been dramatically transformed by the advent of selective *RET*-inhibitors (pralsetinib and selpercatinib), whose impressive and durable response rates shown in clinical trials will inevitably change the treatment paradigm for this molecular subgroup of thyroid cancer (6, 7, 25). In particular, in phase I/II ARROW trial, among the RET-fusion positive TC cohort, including 10 PTC and 1 poorly differentiated thyroid cancer (PDTC), the overall response rate (ORR) was 89%, with a median duration of response (DOR) not reached with a median follow-up of 9.5 months and an estimated 1-year PFS of 81% [6]. Similarly, in phase I/II LIBRET0-001 trial among 19 RET fusion-positive patients, including 13 PTC, 3 PDTC, 2 ATC, 1 Hurthle cell, the ORR was 79%, with activity seen across all histologic types and a 1-year PFS of 64% (7). Notably the efficacy of both pralsetinib and selpercatinib treatment was observed regardless of the number of previous tyrosin kinase inhibitors received, radioiodine treatments, or type of RET mutation/fusion (6, 7).

Indeed, as demonstrated by the present case, in spite of the high histological grade and the coexistence of aggressive *RET* co-mutations, we have witnessed an impressive metabolic and structural tumor response. This response as well as the prolonged clinical benefit confirm the pathogenetic relevance of *RET* rearrangement as a oncogene-addicting driver mutation.

From a clinical perspective, two more issues deserve to be underlined. First, the dramatic response of brain metastases confirms the ability of this class of drugs to cross the blood-brain barrier (BBB). Second, as in other oncogene-driven solid tumors, early metabolic assessment of treatment response may help identify those patients with primarily resistance and select those that will benefit from *RET*-inhibitors.

In this scenario, early recognition of *RET*-rearranged PTC is crucial for several reasons. First of all, given their frequent extrathyroidal extension, multifocal presentation and lymph node involvement, *RET*-rearranged PTC deserve a tailored surgical strategy. Moreover, the identification of those cases with high-grade pathological and molecular features could help clinicians to set up intensive patient surveillance and thus promptly identify those patients who could benefit from early treatment with *RET*-inhibitors. Finally, given the high objective responses achievable, *RET*-inhibitors may be considered in the neoadjuvant setting or before the occurrence of RAI resistance.

In conclusion, the case presented here confirms that early and durable responses can be achieved with *RET* inhibitors in *RET*-rearranged thyroid carcinoma, even in elderly patients with multiple co-morbidities, and in spite of aggressive clinical, pathologic and molecular (*PI3KCA*, *TP53*, and *hTERT* co-mutations) features. It also demonstrates how timely comprehensive molecular testing of those cases wild-type for the common thyroid carcinoma *BRAF V600E*-like and *RAS*-like driver mutations uncovers actionable gene rearrangements that can be targeted by highly selective inhibitors with great potential benefit for the patients.

## Data availability statement

The datasets presented in this study can be found in online repositories. The names of the repository/repositories and accession number(s) can be found in the article/supplementary material.

## Ethics statement

The studies involving human participants were reviewed and approved by local Ethic Committee of S. Orsola-Malpighi Hospital, Bologna (208/2021/Compass/AOUBo). The patients/participants provided their written informed consent to participate in this study.

## Author contributions

MN: conceptualization, writing-original draft; AR: writing – review and editing; GR: writing-original draft; MI: project administration; AG: resources; VM: resources; MF: resources; FN: resources; EL: resources; FM: supervision; ES: resources; AL: formal analysis; TM: formal analysis; MP: supervision, writing – review and editing; DB: conceptualization, writing-original draft, formal analysis, supervision; GT: conceptualization, writing-original draft, resources. All authors contributed to the article and approved the submitted version.

## Funding

The work reported in this publication was funded by the Italian Ministry of Health, RC-2022-2773452.

## Conflict of interest

The authors declare that the research was conducted in the absence of any commercial or financial relationships that could be construed as a potential conflict of interest.

## Publisher’s note

All claims expressed in this article are solely those of the authors and do not necessarily represent those of their affiliated organizations, or those of the publisher, the editors and the reviewers. Any product that may be evaluated in this article, or claim that may be made by its manufacturer, is not guaranteed or endorsed by the publisher.

## Abbreviations

DTC, differentiated thyroid cancer; RAI-R, radioiodine refractory; MAPK, mitogen-activated protein kinase; PI3K, phosphatidylinositol3 kinase; AKT, protein kinase B; NTRK, neurotrophic tyrosine receptor kinase; PTC, papillary thyroid cancer; AF, atrial fibrillation; TSH, thyroid-stimulating hormone; TG, thyroglobulin; TTF-1, Thyroid Transcription Factor- 1; NGS, next-generation sequencing; TB-CT, total body computed tomography; 18F-FDG-PET, 18F-fluoro-deoxy-glucose positron emission tomography; DSV, diffuse sclerosing variant; VAF, variant allele frequencies; BBB, blood-brain barrier.
